# Computational screening and molecular docking of compounds from Traditional Chinese Medicine (TCM) by targeting DNA topoisomerase I to design potential anticancer drugs

**DOI:** 10.1371/journal.pone.0310364

**Published:** 2024-09-12

**Authors:** Mst. Sharmin Sultana Shimu

**Affiliations:** Genetic Engineering and Biotechnology, University of Rajshahi, Rajshahi, Bangladesh; Kafrelsheikh University Faculty of Pharmacy, EGYPT

## Abstract

Each year thousands of people suffer across the globe due to higher cancer incidence and mortality rates. Additionally, the treatment option for cancer patients is also costly, and often cancer drugs suffer from lower efficacy with more side effects. The DNA topoisomerase can function as an established cancer target because Human Topoisomerase (Top1) regulates genetic transcription during the post-mitotic phase and plays a critical role in DNA supercoiling during replication and repair. Therefore, during drug therapy, blocking the Top1 may be crucial for inhibiting the proliferation of cancer cells. Here, the TCM (traditional Chinese medicine) compounds have been screened through the virtual screening. The Chinese medicine library’s virtual screening process made it possible to narrow down the compound list to 29 compounds based on binding energy (-7.1 to -9.3Kcal/mol), while following Lipniski filtering, MM/PB (GB) SA filtering was used to screen the remaining 22 compounds and the top four compounds were chosen based on binding free energy. Here, the four compounds; CID-65752 (T2972: Rutaecarpine), CID-5271805 (T4S2126: Ginkgetin), CID-9817839 (T2S2335: Dehydroevodiamine) and CID-51106 (T3054: Daurisoline) had comparatively higher binding energy of -8.2, -8.5, -8.3 and -8.2 respectively during molecular docking than other compounds. Among these four compounds, no toxic profile of the two screened compounds; CID-5271805 and CID-9817839 was found in ADMET filtering. Moreover, the SASA (solvent accessible surface area), Rg (radius of gyrations), RMSD (root mean square deviation), and RMSF (root mean square fluctuation) profile of the drug-protein complex reveals the stability and rigidity of the compounds in molecular dynamics simulation study. However, these studies need to be validated in experimental approaches to develop more potent and effective cancer drugs.

## 1. Introduction

Cancer poses a high risk threat globally where 10 million deaths as well as 19.3 million newly diagnosed have been reported [1]. A large proportion of cancer patients are from low- and mid-income countries where higher mortality rate is estimated. The significantly higher cost of the treatment as well as the new drug development process resulted in less affordable treatment option [2]. Also, drug resistance towards chemotherapeutic drugs and diverse side effects reduce the efficacy of the cancer drug [3]. Therefore, new cancer drug development with better efficacy and a lesser degree of side effects with affordable options requires complex processes, timing, and funding [4].

The DNA topoisomerase plays a vital role in DNA supercoiling during replication as well as the transcription stage and is important for cell division. It can be targeted for antimicrobial and anticancer drug development processes [5]. It relaxes DNA supercoiling by reducing torsional stress [6] and initiates cleavage of the phosphodiester bond on the DNA strand forming the 3′-phosphotyrosine linkage between the TYR273 [7]. The DNA repair as well as genetic transcription are regulated by Human Topoisomerase (Top1) in post mitotic process. The blockage of the Top1 can be important for the inhibition of cancer cell growth during the drug treatment [8]; and camptothecin [9]. Also, different analogs of the camptothecin [10] have been developed; for example, Topotecan, and Irinotecan [11]. However, they possess multiple side effects [12] and drug resistance [13]. For example, Camptothecin is chemically unstable and associated with the overexpression of ABCB1 and ABCG2 which lead to cross-resistance [14–16].

The computer-aided drug designing and virtual screening procedure dramatically reduce the time and complex procedure by screening readily available or designing new analogs. These approaches can be included in the identification of the drug targets, potential hit identifications from a library [17], and filtering the drug molecules based on pharmacological properties; absorption, distribution, excretion, metabolism, and toxicity [18]. The popular drug-designing approaches include virtual screening, molecular docking, pharmacophore-based designing, molecular dynamics, and QSAR [19].

The structure-based virtual screening procedure is one of the key steps in computer-aided drug designing where screening is conducted by targeting the molecular or biologically relevant target and drugs. This process can be divided into two major groups; ligand-based approaches; where drugs are developed and designed based on the similarity of the drugs or compound; and structure-based virtual screening where the primary focus is given the structure of the protein molecules [20]. The screened database can be also very diverse, natural compounds, synthetic compounds library, and drug repurposing library [21]. Numerous drugs have come into the markets from virtual screening procedures [22]; captopril, ritonavir, tirofibanan, and saquinavir [23].

In this research approach, the Chinese compound list was virtually screened against DNA topoisomerase protein. The best-selected compounds were further screened through MM-GBSA and ADMET filtering to obtain more accuracy. The dynamics simulation approaches were also employed to understand the stable nature of the DNA topoisomerase and compound complexes.

## 2. Material and method

### 2.1. Protein and ligand preparation

The crystal structure of DNA topoisomerase I was retrieved from the Protein Data Bank (PDB:1T8I) [24]. The protein structure was cleaned in Pymol [25] to remove the water molecules, and hetero atoms. The GROMOS 96 43B1 force field was used to optimize the Protein structure [26]. The traditional Chinese medicine Library was prepared from 800 Chinese traditional medicines contain 2390 monomer compounds. This compound list includes various structural types such as flavonoids, alkaloids, terpenoids, and glycosides. Traditional Chinese Medicine library is a powerful library in the fields of anti-cancer, anti-inflammatory, antibacterial, apoptosis, and autophagy research [27].

### 2.2. Molecular docking

The virtual screening calculation was done in DrugRep tools [28] which started with the detecting binding pockets. The binding pockets were set based on binding sites of the protein. The binding pockets were set as ALA498, ALA486, ASP563, HSD367, THR718, ASP533, ARG364, VAL502, THR498, HSD632, LEU485, GLY503, LEU487, GLU495, GLU494, GLU492, THR501, ASN631, PRO368, LY363, ASN366, SER506, CYS630, HET0, ARG488, SER534, ALA715, ALA499, GLU561, PHE361, GLY490, GLY496, ASN491, PHE529, ARG508, LEU629, GLY531, ASP500, LYS532, LYS493, TYR537, ASP562, ARG590, ILE535, GLN633 residue. The selected traditional Chinese medicine library was shown in supplementary material (S1 File). The receptor based screening from DrugRep finds cavity guided screening and searching binding pockets of receptor by using CB-Dock [29] and screens leading compounds with AutoDockVina tools [30]. The custom docking box was set as (12.5 x 5.3 x 18.8) Å in centre where the size (18 x 21 x 28) Å for x,y,z axis respectively. The top screened lead compounds were further selected based on binding energy and subjected to further filtering based on Lipniski rule of five.

### 2.3. Lipniski rule of five filtering

The lipniski rule of five determines the chemical compounds having certain pharmacological and biological properties which may lead to active drug in human. The Lipniski rule states that active drugs follow these mentioned criteria; (1) hydrogen bond donor less than 5, (2) less than 10 hydrogen bond acceptor, (3) less than 500 dalton molecular weight, (4) calculated octanol-water partition coefficient that not exceed 5, (5) number of rotatable bond less than 10. The top screened compounds were further filtered through Lipnsiki rule of five and the compounds which violates more than one rule were excluded [31].

### 2.4. MMGBSA and MMPBSA

The screened compound list was further screened by MM/PB(GB)SA methods where built in docking program was used to generate the ligand binding pose. The calculation and decomposition of binding free energy for each energy minimized protein ligand binding conformation was done. The receptor force field was set as ff19SB with the OPC water model and the ligand force field was set as GAFF2 with truncation radius of 8Å which retained the protein residues within 8Å of all ligand binding pose for rescoring [32].

### 2.5. ADMET

The absorption, distribution, metabolism, excretion, and toxicity (ADMET) were calculated in PKCSM [33]. The canonical smiles of the top four ligand molecules were used as entries for the ADMET calculation.

### 2.6. Molecular dynamics

The YASARA dynamics software package was utilized to perform molecular dynamics simulation [34] where the force field was set as AMBER14. The drug protein complexes were optimized before the simulation along with hydrogen bond orientation and cleaning. The TIP3P solvation model was used to create the cubic simulation cell. The physiological parameter of the dynamics of the drug-protein complex was set as 300K temperature, 7.4 pH, and 0.9% NaCl [35]. The Particle Mesh Ewald (PME) method was applied to calculate the long range electrostatic interaction by a cut off radius of 8 Å. The simulation cell box was set 20 Å larger than the drug-protein complex to allow the free motion. The energy minimization was conducted by the steepest gradient algorithms (5000 cycles) by simulated annealing method. The simulation was performed using an NPT ensemble for relaxation & minimization of the system, where temperature and pressure were maintained at 300 K and 1 atm, respectively, using the Berendsen thermostat and barostat. The time step of the simulation was set as 1.25fs and chemical bond involving the hydrogen bonds was fixed using SHAKE algorithms [36]. The simulation trajectories were saved after 100ps time and extended for 100ns times. The simulation trajectories was utilised to calculate the root mean square deviation (RMSD), root mean square fluctuation (RMSF), radius of gyration (Rg) and solvent accessible surface area [37, 38]. The MM-PBSA was calculated with the MDs trajectories. The MM-PBSA was implemented by using following equation;

Binding Energy = EpotRecept+EsolvRecept+EpotLigand+EsolvLigand−EpotComplex−EsolvComplex

## 3. Results

### 3.1. Virtual screening

The virtual screening of the Chinese medicine library allowed to screen the compound list into 29 compo based on the binding energy where more negative energy indicates more favourable interactions. The top 29 compounds demonstrated binding energy from -7.1 to -9.3Kcal/mol ranges (**Table 1**).

**Table 1:** Virtual screening of 29 compounds from traditional Chinese medicine against DNA Topoisomerase.

Compound ID	Compound Name	Formula	Score	MW	HBD	HBA	RB	NOA	LogP
TN1113	Vincetoxicoside B	C_21_H_20_O_11_	-8.4	448.38	7	8	10	11	0.2
TMA0507	Tomatidine	C_27_H_45_NO_2_	-8.2	415.65	2	1	1	3	6.2
T7602	Theaflavin	C_29_H_24_O_12_	-8.2	564.49	9	10	11	12	-0.6
T3770	Taraxasterol	C_30_H_50_O	-9.3	426.7	1	1	1	1	9.1
T3S0895	Spirostan-3-ol	C_27_H_44_O_3_	-8.6	416.64	1	1	1	3	6.4
T1703	SN38	C_22_H_20_N_2_O_5_	-8.3	392.4	2	5	4	7	3.1
T2972	Rutaecarpine	C_18_H_13_N_3_O	-8.2	287.32	0	2	0	4	3.9
T6S1256	Ruscogenin	C_27_H_42_O_4_	-8.5	430.62	2	2	2	4	4.7
T5783	Rosamultin	C_36_H_58_O_10_	-8.9	650.8	7	8	11	10	3.1
T3778	Pueraria glycoside	C_21_H_20_O_10_	-8.2	432.38	7	8	10	10	-1.1
T5710	Pinocembrin 7-O-beta-D-glucoside	C_21_H_22_O_9_	-8.4	418.39	5	6	9	9	0.9
TN2027	Oxysanguinarine	C_20_H_13_NO_5_	-8.3	347.32	0	1	0	6	4.9
TN6738	Orthosphenic acid	C_30_H_48_O_5_	-8.7	488.7	3	4	4	5	6.2
T5S0788	Oroxin A	C_21_H_20_O_1_0	-8.1	432.38	6	7	10	10	0.0
T3850	Luteolin-7-glucuronide	C_21_H_18_O_12_	-8.3	462.36	7	9	11	12	-0.0
TQ0070	Luteolin-3-O-beta-D-glucuronide	C_21_H_18_O_12_	-8.2	462.36	7	9	11	12	-0.0
T2728	Limonin	C_26_H_30_O_8_	-8.7	470.52	0	3	1	8	1.7
T5S1103	Isoliensinine	C_37_H_42_N_2_O_6_	-7.1	610.75	2	2	11	8	6.3
T6228	Irinotecan	C_33_H_38_N_4_O_6_	-8.2	586.68	1	5	7	10	4.6
T6169	Indirubin	C_16_H_10_N_2_O_2_	-8.1	262.26	2	2	0	4	2.3
TN1733	Hesperetin 7-O-glucoside	C_22_H_24_O_11_	-8.0	464.4	6	7	11	11	0.5
T4S2126	Ginkgetin	C_32_H_22_O_10_	-8.5	566.51	4	6	9	10	4.1
T7600	Fucoxanthin	C_42_H_58_O_6_	-8.5	658.91	2	4	14	6	8.0
T6S0221	Eriocitrin	C_27_H_32_O_15_	-8.2	596.53	9	10	15	15	-1.4
T2S2335	Dehydroevodiamine	C_19_H_16_N_3_O	-8.3	301.34	0	1	0	4	4.1
T3054	Daurisoline	C_37_H_42_N_2_O_6_	-8.2	610.75	2	2	11	8	6.3
T3376	Cynaroside	C_21_H_20_O_11_	-8.4	448.38	7	8	11	11	-0.2
T4S0295	Apigenin 7-glucoside	C_21_H_20_O_10_	-8.1	432.38	6	7	10	10	0.0
TN2435	(-)-Stylopine	C_19_H_17_NO_4_	-8.5	323.35	0	0	0	5	2.9

Here MW, HBD, HBA, RB denotes as molecular weight, hydrogen bond donor, hydrogen bond acceptor, rotatable bond respectively.

Then the Lipniski filtering was applied to screen the compound list where violations of more than one Lipniski rule were considered especially the violations in molecular weight ranges and hydrogen bond donor and number of rotatable bonds were observed. The final 22 compounds after Lipniski filtering were filtered through MM/PB (GB) SA scoring. The MM/PB (GB) SA filtering allows to select top four compounds based on binding free energy (**Table 2**).

**Table 2:** Binding free energy filtering of 22 compounds by MM/PB(GB)SA method.

Compound	PB1	PB3	PB4	GB1	GB2	GB5	GB6	GB7
TN1113	16.66	-7.01	-11.95	-31.17	-24.33	-23.24	-12.15	-17.92
TMA0507	22.16	-9.44	-13.17	-31.62	-27.98	-27.48	-13.16	-28.58
T3770	22.31	-2.04	-10.94	-32.52	-28.35	-27.71	-9.71	-30.21
T3S0895	17.05	-2.56	-10.52	-29.05	-25.08	-24.31	-11.02	-24.88
T1703	22.86	-4.92	-7.82	-33.81	-28.56	-27.57	-8.46	-27.01
T2972	2.23	-16.23	-21.72	-35.91	-31.96	-31.01	-24.39	-32.38
T6S1256	12.73	-2.06	-13.65	-27.71	-26.69	-26.1	-23.33	-19.72
T3778	12.41	-9.61	-15.42	-32.84	-25.68	-24.37	-14.23	-20.3
T5710	9.22	-13.36	-16.52	-31.13	-25.88	-25.3	-17.3	-22.89
TN2027	17.84	-6.47	-21.39	-26.55	-17.2	-26.2	-2.68	-26.61
TN6738	12.18	-12.41	-19.97	-37.58	-31.84	-30.95	-15.72	-31.03
T5S0788	12.75	-6.47	-14.24	-31.9	-24.69	-23.16	-12.44	-16.95
T2728	16.38	-9.22	-16.8	-31.47	-38.90	-28.7	-16.28	-31.26
T6228	12.24	-8.76	-11.89	-27.6	-31.24	-26.71	-12.21	-29.90
T6169	1.07	-14.51	-19.18	-32.29	-28.92	-28.07	-20.98	-28.09
TN1733	11.72	-3.86	-13.01	-30.38	-24.27	-23.36	-14.59	-19.11
T4S2126	15.81	-9.44	-21.28	-42.64	-36.16	-35.27	-18.52	-43.12
T2S2335	-2.07	-23.46	-26.3	-38.46	-34.77	-33.78	-28.62	-35.01
T3054	2.95	-18.92	-31.09	-51.6	-44.99	-43.95	-29.5	-45.34
T3376	7.79	-8.81	-17.36	-34.44	-27.84	-26.7	-18.34	-22.54
T4S0295	12.87	-11.59	-17.63	-29.89	-24.46	-23.99	-16.56	-22.03
TN2435	16.77	-8.65	-16.76	-36.19	-23.43	-24.11	17.88	-29.91

### 3.2. Binding interaction of the top compounds

**Fig 1:**
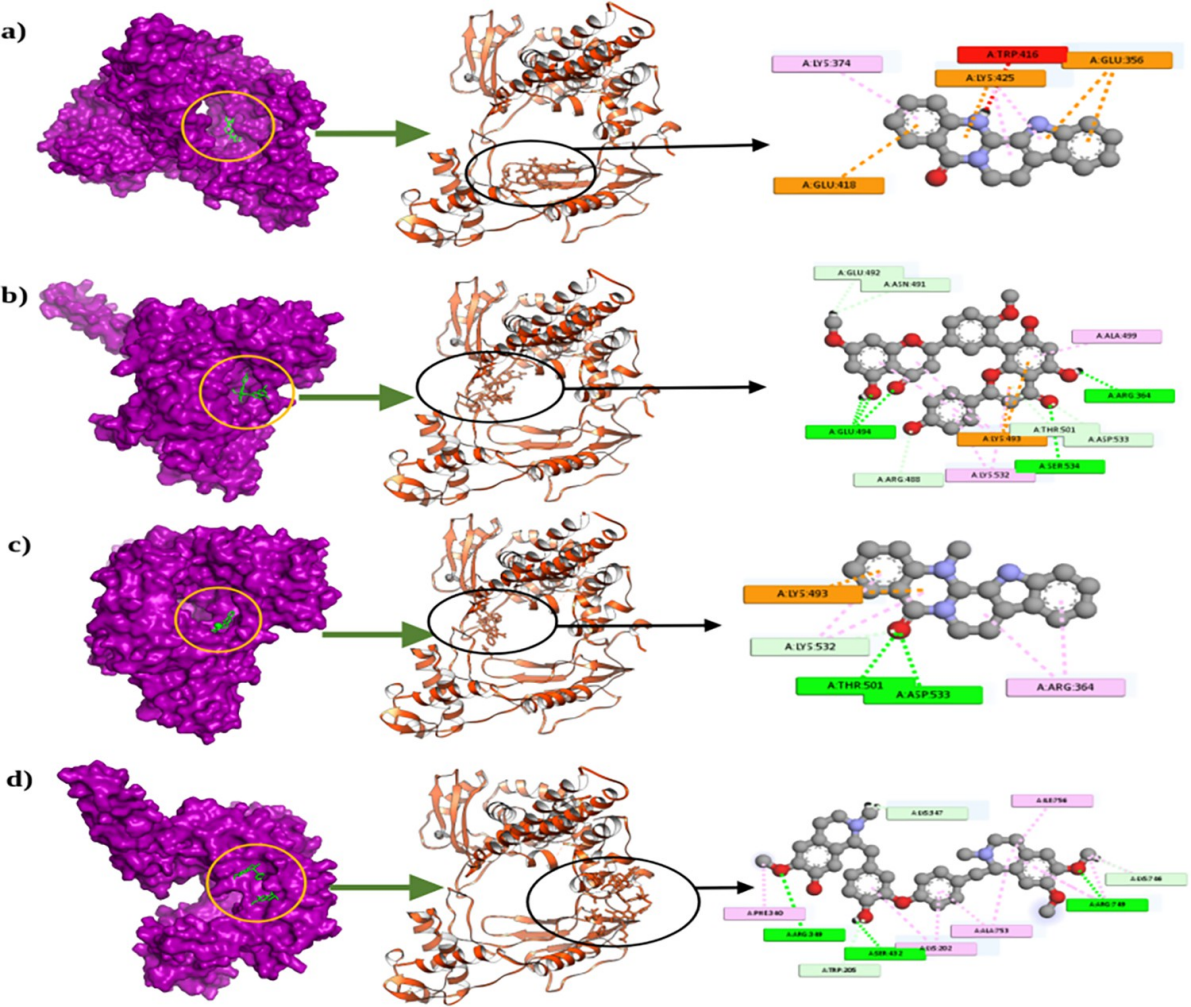
Molecular docking study of DNA topoisomerase I with top four active compounds from Traditional Chinese Medicine. Here, a) indicates the surface view, cartoon shape, and 2D view of CID- 65752 and DNA topoisomerase I; b) indicates the interaction of CID-5271805 and DNA topoisomerase I; c) represents three different view of CID- 9817839 and DNA topoisomerase I; d) represents three different view of CID- 51106 and DNA topoisomerase I.

The top 4 compounds and their binding residues while interacting with the target topoisomerase protein were retrieved from Pymol and Discovery Studio Software Package (**Fig 1**).

The CID-65752 and DNA topoisomerase complex had multiple Pi-Cation interactions at LYS525, GLU356, and GLU418 position and also one Pi-alkyl interaction was observed at LYS374 position. The CID-5271805 and DNA Topoisomerase complex was stabilized by five hydrogen bonds at GLU494, SER534, ARG364, ARG488, and THR501 residues. Also, this complex had one pi-cation bond at Lys493 and one pi-alkyl interaction at LYS532 residues. The CID-9817839 and protein complex showed two hydrogen bonds at ASP533, THR501, and one Pi-cation at LYS493, one alkyl at ARG364 and one Pi-Alkyl at LYS532 residues. The CID-51106 and DNA topoisomerase complex had five hydrogen bonds at ARG349, SER432, ARG749, LYS746, and LYS347 residues. It also had one alkyl interaction at ILE756, and two Pi-Sigma interaction at ALA753, and LYS202 (**Table 3**).

**Table 3:** The binding interactions of the top 4 compounds where hydrogen bond, Alkyl bond, and Pi-Alkyl interactions were observed.

Complex	Residue	Interaction	Distance(Å)
CID-65752 (T2972)	LYS425	Pi-Cation	4.70
	GLU356	Pi-Cation	4.49
	GLU418	Pi-Cation	4.79
	LYS374	Pi-Alkyl	4.47
CID-5271805 (T4S2126)	GLU494	Hydrogen	2.03
	SER534	Hydrogen	2.65
	ARG364	Hydrogen	2.29
	ARG488	Hydrogen	3.77
	THR501	Hydrogen	2.88
	LYS493	Pi-Cation	4.56
	LYS532	Pi-Alkyl	4.03
CID-9817839 (T2S2335)	ASP533	Hydrogen	2.09
	THR501	Hydrogen	2.19
	LYS493	Pi-Cation	4.07
	ARG364	Alkyl	5.05
	LYS532	Pi-Alkyl	5.26
CID-51106 (T3054)	ARG349	Hydrogen	2.78
	SER432	Hydrogen	1.80
	ARG749	Hydrogen	2.30
	LYS746	Hydrogen	3.56
	LYS347	Hydrogen	3.20
	ILE756	Alkyl	4.85
	ALA753	Pi-Sigma	3.92
	LYS202	Pi-Sigma	3.70
	PHE340	Pi-Alkyl	4.75

### 3.3. ADMET

The water solubility of compounds reflects the solubility of the molecules at 298K temperature. The lipid-soluble ones are less well absorbed than the water-soluble ones. The T4S2126 and T2S2335 had water solubility ranges from -2.92 to -3.6. The higher Caco2 permeability is measured as a value more than 0.90 where T2S2335 and T2972 had more Caco2 permeability compared to other compounds. The ability of the drug molecules to pass the blood-brain barrier is a crucial parameter to alleviate the side effects and toxicities to enhance drug efficacy. The BBB permeability >0.3 indicates the readily cross the blood-brain barrier and logBB <-1 indicates the poor distribution to the brain. The T2972 and T2S2335 had more logBB values than readily distributed thresholds (**Table 4**). The AMES and hepatotoxicity indicate that T2972 and T3054 had a probability of being toxic, and were excluded for further downstream analysis.

**Table 4:** Absorption, distribution, metabolism, excretion and toxicity of the selected compounds.

ID	Water Solubility	Caco2 Permeability	BBB Permeability	AMES Toxicity	Oral Rat Acute Toxicity	Hepatotoxicity
T2972	-3.459	1.26	0.699	Yes	2.431	Yes
T4S2126	-2.92	-0.84	-1.84	No	2.733	No
T2S2335	-3.6	1.74	0.353	No	2.12	No
T3054	-3.652	0.567	-1.047	Yes	2.463	Yes

Here the water solubility, Caco2 permeability, BBB permeability, oral rat acute toxicity were calculated as log mol/L, log Papp in 10–6 cm/s, log BB, mol/kg units respectively.

### 3.4. Molecular dynamics simulation

**Fig 2:**
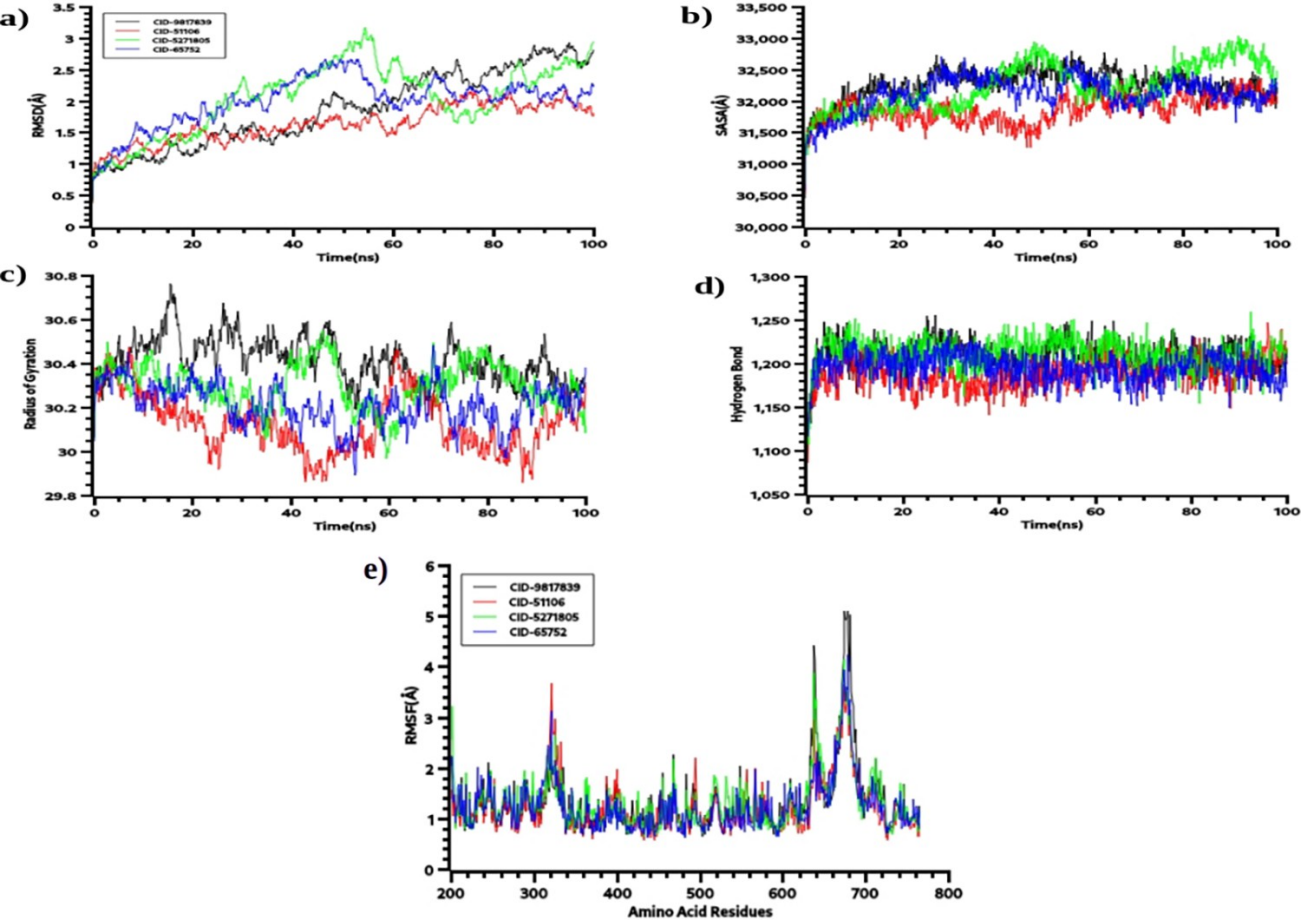
The molecular dynamics simulations study of the DNA topoisomerase I. a) the root mean square deviations, b) solvent accessible surface area, c) hydrogen bond, d) radius of gyrations, e) the root mean square fluctuations of the complexes.

The RMSD or root mean square deviation of the simulation trajectories defines the stable nature of the drug-protein complex. **Fig 2A)** indicates that initially, the drug-protein in the dynamics system had a higher RMSD trend which represents the more mobile nature of the drug-protein system at the initial phase of the simulation. Every complex became stable after 30ns and maintained the stability of the complex till 100ns periods. The CID-9817839, and CID-5271805 complexes had higher RMSD when compared to other complexes which indicate comparatively less stability than other complexes. The overall RMSD of the compounds were below 2.5Å which defines the overall more stable nature of the complexes. This RMSD trend also demonstrates the less flexible nature of the complex.

The SASa or solvent-accessible surface area of the drug-protein complex from the simulation trajectories represents the surface area changes while higher SASA relates with the extension of the surface area and lower SASA defines the truncated nature of the complexes. **Fig 2B)** indicates the CID-5271805 complex had a higher SASA value which indicates the complexes had a loose packaging system during the simulation periods. As a result, the complexes had a more flexible nature while interacting with the complexes. The rest of the complexes had stable SASA trends till 100 ns periods.

The radius of gyration of the complexes indicates the mobile nature of the complexes. **Fig 2C)** indicates that the CID-9817839 and CID-5271805 complexes had higher Rg profiles. This higher Rg profile of those complexes shows the higher flexible nature of the complex. The hydrogen bond of the complexes defines the stability of the complexes where **Fig 2D)** indicates the complexes had less fluctuating hydrogen bonding trend during simulation. The hydrogen bond trajectories were stable across the simulation time. The RMSF or root mean square fluctuation of the amino acid residues of the protein complex represents the changes and flexible nature of the amino acid residues in a protein complex. **Fig 2E)** indicates the complexes had lower RMSF than 2.5Å except few residues. The lower RMSF profile for the maximum residues indicates the stable nature of the complexes.

**Fig 3:**
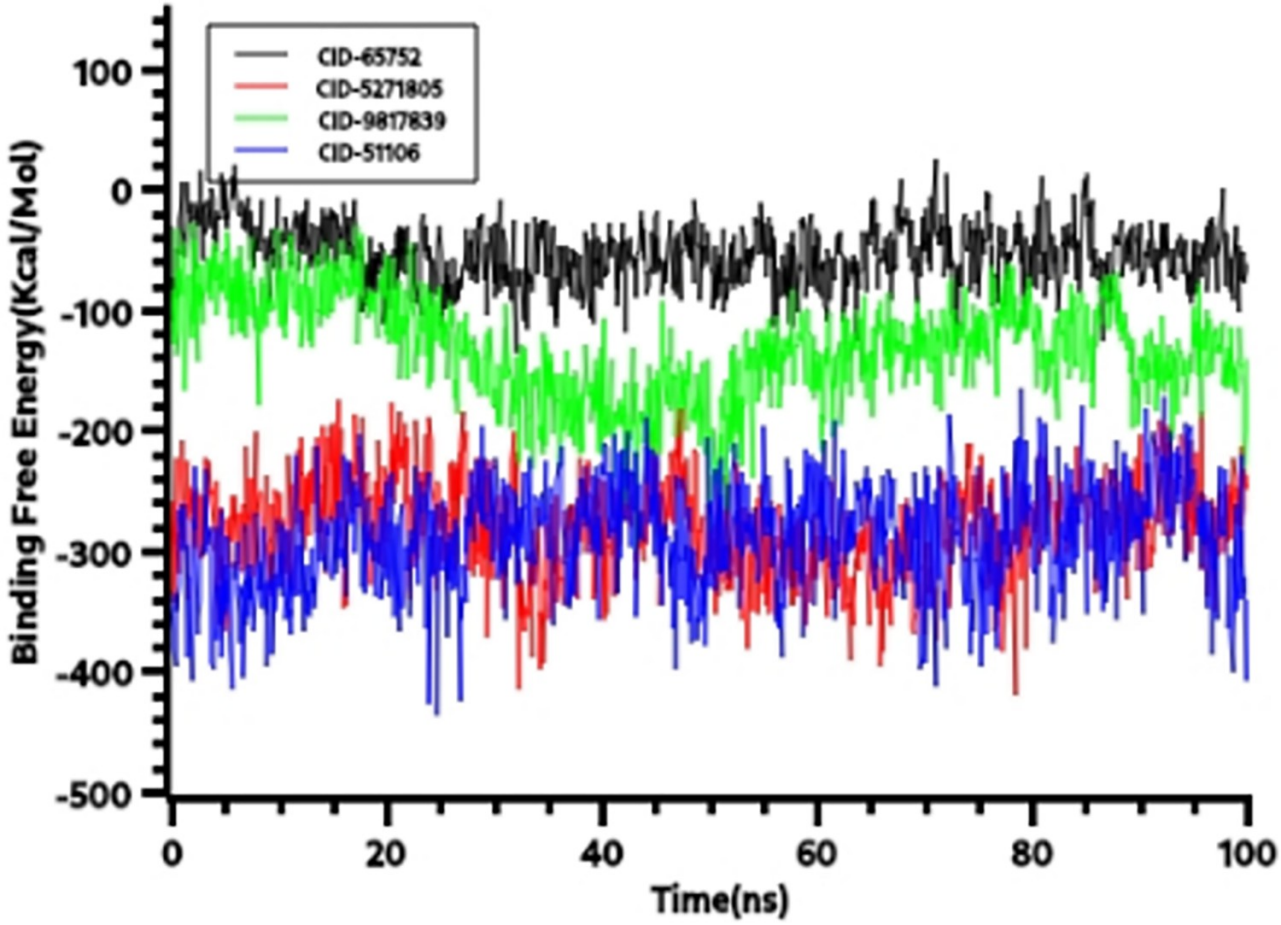
The binding free energy from YASARA MM-PBSA where the positive energy indicates more favorable binding. The CID-65752 had better binding than other complex.

Also, binding free energy was calculated from the MM-PBSA approach from simulation trajectories of YASARA. The more positive energy from MM-PBSA from the YASARA algorithm indicates more favourable binding. **Fig 3** indicates that CID-65752 had higher energy in MM-PBSA compared to other complex which indicates more favourable binding of that complex.

## 4. Discussion

The computer-aided drug design illustrates a new era by providing lesser time and cost as well as a labouring process which makes this method more feasible. It has become an important tool for designing drug analogues and new drug development. Also, it provides a combination of experimental and computational frameworks to accelerate the drug discovery process. The integrated virtual screening, molecular docking, ADMET, and dynamics simulation approaches can aid in shortlisting the most biologically relevant and active compounds [39–42]. The computer-aided drug designing can target specific molecules by using the structural information’s and the nature of their binding pattern [43–45].

Traditional Chinese medicine (TCM) plays an important role in the treatment of patients for long periods in China and Asian countries. The rich phytochemical content of those plant species; tannin, alkaloid, flavonoids, and other content can be utilized to use drug development with higher efficacy [46]. To date multiple drugs have been developed from TCM for example; artemisinin which is found majorly in Artemisia carvifolia known to treat acute leukemia [47], chemotherapeutic drug taxol [48], cardio-protective drug Danshensu [49], and salvicine for treating the solid tumor. Although recent studies show that some TCM compounds have toxic effects [49], systemic investigation of TCM compounds requires to develop of therapeutic drugs from TCM compounds with fewer side effects and toxicity [50]. The virtual screened compounds in this study consist of rich diversity of flavonoids, alkaloids, terpenoids, and glycosides [51]. These herbal drugs possess multiple evidence of uses in drug development and design with less toxicity [52].

The Lipinski rule of five plays an important role in determining drug-likeness screening. It helped to additionally screen 7 compounds in the virtual screening process. The CID- 65752 and DNA topoisomerase complex interaction reveals the interaction at GLU356 and LYS374 residues where residue contact was also observed for the crystal structure of DNA topoisomerase. The multiple interactions at active site points indicate the strong binding nature of the complex. This compound is also reported to inhibit hyperplasia in rat model [53], also improve cognitive function by improving mitochondrial function [54], treatment of liver diseases [55], and function against pathogenic fungi [55]. The CID-5271805 and DNA Topoisomerase had multiple interactions at ARG364, ARG488, THR501, LYS493, and LYS532 residues where the crystal structure had similar interaction sites. The CID-5271805 also possesses a diverse range of effects on the cellular system; anti-inflammatory activity and anti-apoptotic effect [56], promotes M2 polarisation of microglia through PPARγ signalling pathway, and inhibits neuroinflammation [56]. The CID-9817839 and protein complex had similar interactions at ASP533, THR501, LYS493, ARG364, and LYS532 residues like crystal structures. The CID-9817839 is involved in inhibiting metastasis [57], suppressing the inflammatory response in rat and human systems [58], improving stress-induced memory impairment [59], and enhancing cognitive function. The CID-51106 and DNA topoisomerase complex had two interaction points similar to the crystal structure at LYS746 and LYS347. Also, the compound had multiple hydrogen bonds, where the interaction or presence of multiple hydrogen bonds indicates the strong binding nature of the complexes. The CID-51106 inhibits esophageal squamous cell carcinoma growth in vitro and in vivo conditions [60], inhibits tumor angiogenesis [61], and suppresses lung cancer tumorigenesis [62].

## 5. Conclusion

This study represents the virtual screening of the TCM compounds against the DNA topoisomerase to design cancer drugs. The four compounds CID-65752 (T2972: Rutaecarpine), CID-5271805 (T4S2126: Ginkgetin), CID-9817839 (T2S2335: Dehydroevodiamine), and CID-51106 (T3054: Daurisoline) had higher scoring in docking and binding free energy filtering. The molecular dynamics study of the complex reveals that the complexes had exhibited stability across the simulation trajectories and RMSD, RMSF, SASA, and Rg. The lower toxicity and drug-likeness of the compounds were also revealed in ADMET screening, where T2972 and T3054 had been excluded due to the probability of being toxic. Finally selected two screened compounds; CID-5271805 (T4S2126: Ginkgetin) and CID-9817839 (T2S2335: Dehydroevodiamine) had also important roles in cellular activity and development other drugs development that have been represented in the discussion. This study represents that the TCM compound can be used to design and develop the cancer drug, however, validation from the in vitro and in vivo conditions is required for further assessment.

## Supporting information

S1 File (SDF)
